# C-Tb skin test to diagnose *Mycobacterium tuberculosis* infection in children and HIV-infected adults: A phase 3 trial

**DOI:** 10.1371/journal.pone.0204554

**Published:** 2018-09-24

**Authors:** Henrik Aggerbeck, Morten Ruhwald, Søren T. Hoff, Bettine Borregaard, Elizabeth Hellstrom, Mookho Malahleha, Mirna Siebert, Mashra Gani, Vincent Seopela, Andreas Diacon, Madeleine Lourens, Peter Andersen, Keertan Dheda

**Affiliations:** 1 Department of Vaccine Development, Statens Serum Institut, Copenhagen, Denmark; 2 Department of Infectious Diseases Immunology, Statens Serum Institut, Copenhagen, Denmark; 3 Be Part Yoluntu Centre, Paarl, South Africa; 4 Setshaba Research Centre, Pretoria, South Africa; 5 Tiervlei Trial Centre, Cape Town, South Africa; 6 Global Clinical Trials, Port Elizabeth, South Africa; 7 Synexus Stanza Bopape Clinic, Pretoria, South Africa; 8 TASK, Cape Town, South Africa; 9 UCT, Cape Town, South Africa; National Institute for Infectious Diseases (L. Spallanzani), ITALY

## Abstract

**Background:**

C-Tb, an ESAT-6/CFP-10-based skin test, has similar sensitivity for active TB compared to tuberculin skin test (TST) and QuantiFERON-TB-Gold-In-Tube (QFT). However, data are limited in children and HIV-infected persons.

**Methods:**

Asymptomatic South African contacts <5 years (n = 87; HIV-uninfected), or symptomatic individuals of all ages presenting to clinics with suspected TB (n = 1003; 30% HIV-infected) were recruited from eight South African centres. C-Tb and TST were allocated to either forearm double blinded. Samples for QFT were collected in parallel, and test-positivity rates were compared.

**Results:**

In participants with microbiologically confirmed TB (n = 75; 45% HIV-infected) sensitivity of C-Tb, TST and QFT were similar (72% versus 75% versus 73%; p>0.5). All 3 tests had similar positivity rates in HIV-infected participants with active TB, however, positivity rates were reduced when CD4 counts were <100 cells/μL. In participants where active TB was excluded (n = 920), C-Tb (41%), TST (43%), and QFT (44%) also had similar test-positivity rates. Among asymptomatic contacts aged below five, 32% (28/87) tested positive with C-Tb and 32% (28/87) with TST (concordance 89%). Overall, C-Tb and TST showed a similar safety profile.

**Conclusion:**

C-Tb was safe and showed similar test-positivity rates, compared to TST and QFT, in children and HIV-infected persons with active or latent *M*. *tuberculosis* infection. These data inform the utility of C-Tb in clinical practice.

**Trial registration:**

ClinicalTrials.gov NCT01642888.

EudraCT 2011-005078-40.

## Introduction

In contrast to active TB, which can be microbiologically proven, latent *M*. *tuberculosis* infection (LTBI) can only be inferred indirectly by the detection of an antigen-specific immune response, either in the skin through the tuberculin skin test (TST) using purified protein derivative (PPD), or *in vitro* using a blood-based interferon-γ release assay (IGRA) [1,2].

One strategy to decrease the burden of TB in low and high TB-burden settings is to systematically test those with LTBI, at highest risk of progressing to active disease, but are willing to accept treatment. Risk groups include people living with HIV, and adult and childhood contacts of active cases of pulmonary TB (PTB) [1,3–5]. However, there are several drawbacks of the currently approved tests. The TST may give false-positive reactions from cross-reactivity with non-tuberculous mycobacteria and previous Bacillus Calmette–Guérin (BCG) vaccination especially if BCG is administered after infancy [6–8]. In HIV co-infection, the cut-point may need adjustment to mitigate for lower sensitivity. More recently, the IGRAs QuantiFERON-TB Gold In Tube (QFT; Qiagen, Hilden, Germany) and T-SPOT.*TB* (Oxford Immunotec, Abingdon, UK) have become routine tests to diagnose presumed *M*.*tb* infection in low-burden countries, either alone or in combination with TST [9,10]. The IGRAs are more specific and require only a single visit for phlebotomy. However, a second visit may often be needed to clarify the result, complete symptom screening for active TB, and to start LTBI treatment. The IGRAs also have several drawbacks, including the need for complex laboratory and sample-transport infrastructure, higher costs, within person variability, and a high proportion of indeterminate results in advanced HIV and very young children [11].

C-Tb (Statens Serum Institut, Copenhagen, Denmark) is a highly specific skin test for the diagnosis of LTBI designed to address some of the drawbacks of TST and IGRAs [12]. C-Tb is applied and read in the same way as TST, but is based on the antigens ESAT-6 and CFP-10 that are also included in the IGRAs. Due to high specificity, C-Tb uses a universal 5 mm cut-point induration irrespective of the status of BCG, HIV, or both [13]. In a recent contact tracing trial that included groups with various degrees of exposure to cases of PTB, C-Tb test-positivity rates were concordant with increasing exposure to TB, and were very highly concordant with QFT [14].

However, little is known about the value of C-Tb in other risk groups including young children and persons living with HIV [3,15,16]. To address this question, we conducted a phase 3 trial that included participants of all ages (with varying HIV status). Participants included those with suspected active TB, asymptomatic children <5 years old that were close contacts of cases with active PTB, and healthy older children served as community-based control group. As there is no microbiological gold standard for LTBI, as in previously published evaluation studies [11,17], sensitivity was determined in active TB [13], with the presumption that sensitivity would be similar or possibly better in LTBI (given that active TB is an immunosuppressive state).

## Materials and methods

### Trial design and participants

**Fig 1:**
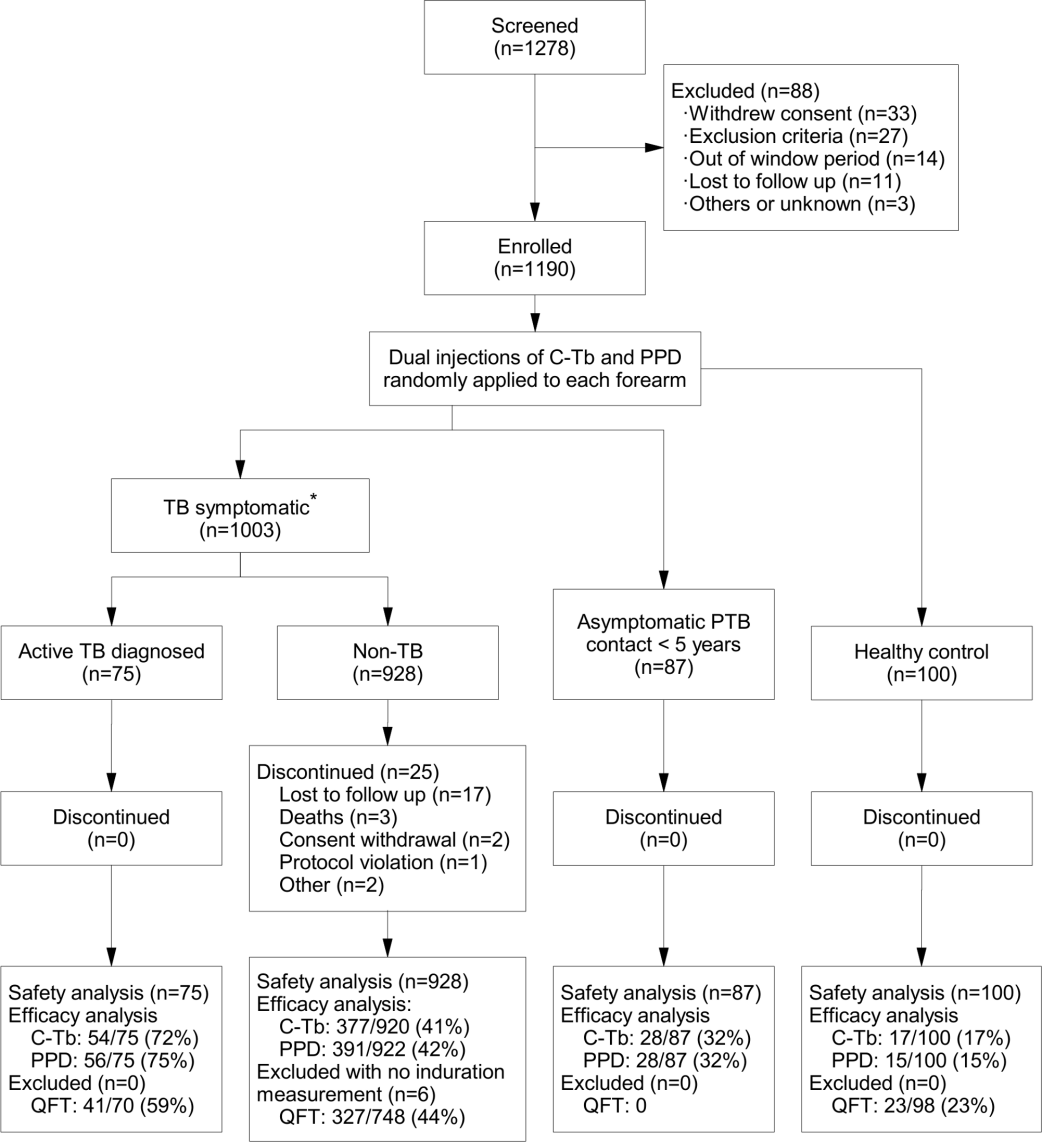
Overview of the trial participants. *These were clinic attendees that on follow-up segregated into those with microbiologically confirmed active TB and non-TB participants. Efficacy data are presented as number of test positives/n (%).

The trial was a double-blind, split-body design with randomized injections of C-Tb versus TST in either forearm. The trial was conducted in eight South African sites from September 05, 2012 to September 30, 2014. 1003/1190 of those enrolled were cases with suspected TB (0–65 years; 30% HIV-infected), while the remainder comprised 87 asymptomatic PTB contacts aged below five, and 100 healthy controls (5–11 years old; Fig 1). The latter was to infer the impact of recent exposure on skin test responses using ‘supposedly’ non-exposed children (no known contact but within a TB endemic environment where the force of infection is high) as a reference, and to complete safety evaluations over a range of childhood subgroups (children with active TB, asymptomatic contacts of active cases, and healthy controls that did not have a definitive history of exposure).

Those with suspected TB between 0 and 65 years of age presented to primary care clinics with TB symptoms (online data supplement S1 Table). These individuals, after investigation and follow-up for 28 days, were classified as either active TB cases (culture or Xpert MTB/RIF positivity served as the reference standard) or non-TB. Participants with clinical symptoms of TB but with a negative microbiology were included in the non-TB group, because only microbiologically confirmed TB was regarded gold standard to evaluate the sensitivity of the tests. The asymptomatic children aged <5 years were close contacts of a smear positive PTB case defined as more than 6 h contact per day for at least five days. The trial staff also recruited a healthy control group of children with no known contact with people infected with *M*.*tb* and no symptoms or signs of active TB (as outlined above). Subjects with confirmed active TB or AIDS defining diagnosis at screening were excluded.

### Trial procedures

Identical appearing vials of C-Tb and PPD RT 23 (both Statens Serum Institut, Copenhagen, Denmark, (since 2017 PPD RT 23 is a product held by AJ Vaccines) labelled left or right were randomized in blocks of 10, and 0.1 mL of each agent was randomly injected in separate arms immediately one after the other according to the Mantoux technique as previously described [14]. Induration responses for C-Tb and TST were read 48–72 h after intradermal injection transversely to the long axis of the forearm with the ballpoint method and a ruler, and documented with digital images. Indurations ≥1 mm defined a ‘responder’. Samples for QuantiFERON-TB-Gold-In-Tube (Qiagen, Hilden, Germany) were collected prior to the skin tests. The QFT was performed by PathCare (Goodwood, South Africa) according to the manufacturer’s instructions. BCG status was determined according to vaccination cards and the presence of BCG scars. HIV-infection was diagnosed by two alternative positive rapid tests, or one positive rapid test and a confirmatory ELISA. To limit the amount of blood taken from those aged <5 years old (as per ethical approval and guidelines), no blood samples for haematology, biochemistry, QFT or HIV status were collected. The HIV-test result obtained immediately after birth was used.

### Safety assessment

Local and systemic adverse events (AE) were assessed by the trial staff through medical assessments 30 minutes after intradermal injection and at the follow-up visits. Diaries were provided for recording of all AEs experienced until Day 28. Injection site reactions (ISRs) were assigned to the respective agent, whereas systemic AEs were assigned to both agents.

### Objectives and outcomes

The primary trial objective was to assess the performance of C-Tb in children and HIV-infected persons and to confirm the safety profile. The primary outcome was the test-positivity rate defined by indurations ≥5 mm for C-Tb and 5 or 15 mm for TST (5 mm if HIV-infected) [10,12,13]. Secondary outcomes were comparisons of C-Tb, TST, and QFT test positivity. As there is no gold standard for diagnosing LTBI, we provided comparative test positivity data for C-Tb, QFT, and TST.

### Statistical analysis

Given the lack of a microbiological definition of LTBI, sensitivity was determined in participants with active TB. In those without active TB (non-TB), C-Tb test-positivity rates were compared to QFT and TST. Differences in number of test positives were assessed by McNemar’s test for marginal heterogeneity, and in unpaired analyses with Fisher’s exact test or χ^2^ test. Agreement between tests was assessed with Cohen’s κ coefficient. Given that this trial addressed patient safety and used within-person randomisation the results have been presented within the context of the Consort Guidelines. Further details are provided in the online supplement.

### Ethics

The trial was conducted according to the principles expressed in the Declaration of Helsinki. Written informed consent form were obtained from adult participants and from parents or legal guardians of children, with additional assent form provided in older children. The trial was approved by Pharma-Ethics (No. 12024740), University of Cape Town Human Research Ethics Committee (No. 222/2012), and the Medicines Control Council (No. 20120120). The trial was part of an agreed Paediatric Investigation Plan, which is in accordance with EU regulations.

## Results

### Participants and final diagnosis

During the observation period of 28 days following skin testing, active TB was confirmed among 75/1003 (7%) with TB symptoms, while 928/1003 (93%) had no microbiological evidence of active TB (non-TB but this included 44 participants with symptoms who were test negative but empirically treated for TB).

Out of the 928 non-TB cases, two did not receive C-Tb, and six participants were either lost to follow-up, withdrew consent, or died; thus no induration measurements were recorded in these participants. None of the three reported deaths were related to the skin tests. Among the enrolled participants ≥5 years old, 38 (4%) had missing QFT result, and among the remaining 916, 131 (14%) were indeterminate (all due to low IFN-γ levels in the positive control).

Both genders were equally distributed with the median age of 17 years (Table 1). The majority of the trial population was of Black African descent (55–87%). The healthy control group inadvertently included participants only of mixed race (91%) and of European descent (9%). There was almost full BCG vaccination coverage among young children. HIV-infection was mainly observed in adults. Participants with unknown HIV status were all ≤5 years of age. Median CD4 count in HIV-infected adults was 314 cells/**μ**L (IQR 164–502 cells/**μ**L).

**Table 1:** Demographics of the trial population.

		ALL	Symptomatic		Asymptomatic	Healthy control
			Diagnosed with active TB*	Non-TB	PTB contact	
**Trial participants**		1190	75	928	87	100
**Age (years)**						
	Median (range)	17 (0–65)	32 (0–62)	25 (0–65)	2 (0–4)	8 (5–11)
	<5	236 (20)	2 (3)	147 (16)	87 (100)	0 (0)
	5–17	366 (31)	13 (17)	253 (27)	0 (0)	100 (100)
	≥18	588 (49)	60 (80)	528 (57)	0 (0)	0 (0)
**Sex**						
	Male	589 (49)	40 (53)	465 (50)	43 (49)	53 (53)
	Female	601 (51)	35 (47)	463 (50)	44 (51)	47 (47)
**Ethnicity**						
	African descent	909 (76)	55 (73)	806 (87)	48 (55)	0 (0)
	Other	291 (24)	20 (27)	122 (13)	39 (45)	100 (100)
**BCG status**						
	Vaccinated	882 (74)	45 (60)	655 (71)	86 (99)	96 (96)
	Not vaccinated	264 (22)	25 (33)	234 (25)	1 (1)	4 (4)
	Unknown	44 (4)	5 (7)	39 (4)	0 (0)	0 (0)
**HIV**						
	Uninfected	730 (61)	40 (53)	566 (61)	24 (28)	100 (100)
	Unknown	161 (14)	1 (1)	97 (10)	63 (72)	0 (0)
	Infected	299 (25)	34 (45)	265 (29)	0 (0)	0 (0)
**CD4 (HIV-infected)** ^†^						
	<100	40 (14)	12 (35)	28 (11)	N.A.	N.A.
	≥100	246 (86)	22 (65)	224 (89)	N.A.	N.A.

Data are presented as n, median (range) or n (%).

*Microbiologically confirmed during the trial after skin testing.

^†^13 missing CD4 count.

### Test performance

#### Overall

C-Tb showed a positivity rate of 40% (95% CI 38–43%, n = 1182), similar to the positivity rate of TST (41% [39–44%], n = 1184, p = 0.3456) and the results were concordant in 84% of the participants. QFT was not done in children <5 years old; for those ≥5, QFT showed a positivity rate of 43% ([40–46%], n = 916), including 131 (14%) indeterminate results. C-Tb and QFT showed no significant difference among those subjected to dual testing and the results were concordant in 624 (83%) of the 750 participants with a determinate test result (p = 0.1987). The impact of age on test readouts is outlined in the online supplement.

### Sensitivity, positivity rates and the impact of HIV

Based on the 75 cases with microbiologically confirmed active TB, C-Tb showed a sensitivity of 72% (95% CI 61–81%), similar to the sensitivity of TST (75% [64–83%], p = 0.8026) and results were concordant in 59 (79%) of the participants (Table 2, S2 Table). Forty-five percent (34/75) of the confirmed cases were co-infected with HIV, even though HIV-infection only constituted 30% (299/1003) of the participants with TB symptoms (Table 1). The sensitivity of QFT was influenced by five missing results and 14/70 (20%) indeterminate results, mainly (9/14, 64%) in the HIV-infected group (S2 Table). Seventy-one percent (10/14) of the participants with indeterminate QFT result tested positive with C-Tb, and 79% (11/14) tested positive with TST. Based on those tested, QFT showed a sensitivity of 59% (41/70), increasing to 73% (41/56%) if indeterminate results were excluded (Table 2, S2 Table).

**Table 2:** Sensitivity of C-Tb versus TST (top) and QFT (bottom) in microbiologically confirmed active TB cases.

	All	HIV-uninfected	HIV co-infected
**C-Tb versus TST**			
**N**	75	41	34
**C-Tb pos. (%)**	72.0 (60.9–81.0)	75.6 (60.5–86.4)	67.7 (50.8–81.0)
**TST pos. (%)**	74.7 (63.7–83.2)	73.2 (57.9–84.4)	76.5 (59.8–87.8)
p-value*	0.8026	1.0000	0.3711
**C-Tb versus QFT**			
**N**	70	38^†^	32^‡^
**C-Tb pos. (%)**	70.0 (58.4–79.5)	73.7 (57.8–85.2)	65.6 (48.2–79.7)
**QFT pos. (%)**	58.6 (46.9–69.4)	68.4 (52.5–81.0)	46.9 (30.9–63.6)
p-value*	0.1175	0.7518	0.1138

Data are presented as % (95% CI). Cut-point for TST was 5 mm in HIV-infected individuals and 15 mm in HIV-uninfected.

*McNemar’s test. In an intention to diagnose principle, QFT indeterminate results were included as negative.

^†^3 missing QFT. 5/5 QFT indeterminate results were C-Tb positives.

^‡^2 missing QFT. 5/9 QFT indeterminate results were C-Tb positives.

In symptomatic non-TB participants, C-Tb showed a positivity rate of 41% (39–44%), similar to the rate of TST (43% [39–46%], p = 0.298) and QFT (44% [40–47%], p = 0.1105), and with a concordance of 83% and 79%, respectively (Table 3, S3 Table). All three tests showed similar positivity rates in HIV-uninfected individuals. However, application of a 5 mm cut-point for TST in HIV-infected participants was associated with a significantly higher test positivity rate of 40% (34–46%) compared to C-Tb (34% [29–40%], p = 0.0180) and QFT (26% [21–32%], p = 0.0004). QFT showed a high number of indeterminate results (111/741 [15%]), mainly 68/111 (61%) in HIV-infected participants (S3 Table).

In the 245 HIV-infected non-TB participants with available QFT test results, 81 (33%) tested positive with C-Tb and only 65 (27%) tested positive with QFT (p = 0.0412, Table 3, S3 and S4 Tables). Excluding indeterminate results, QFT positivity rate increased from 27% (65/245) to 37% (65/177), which was similar to the positivity rate of C-Tb (37% [66/177], S3 Table).

**Table 3:** Test positive results of C-Tb versus TST (top) and QFT (bottom) in non-TB participants.

	All	HIV-uninfected	HIV- infected
**C-Tb versus TST**			
**N**	920	658^†^	262
**C-Tb pos. (%)**	41.0 (37.8–44.2)	43.6 (39.9–47.4)	34.4 (28.9–40.3)
**TST pos. (%)**	42.5 (39.3–45.7)	43.5 (39.7–47.3)	40.1 (34.3–46.1)
p-value*	0.2980	1.0000	0.0180
**C-Tb versus QFT**			
**N**	741	496^‡^	245^§^
**C-Tb pos.**	46.6 (43.0–50.2)	53.2 (48.8–57.6)	33.1 (27.5–39.2)
**QFT pos.**	43.7 (40.2–47.3)	52.2 (47.8–56.6)	26.5 (21.4–32.4)
p-value^*^	0.1105	0.6935	0.0412

Data are presented as % (95% CI). Cut-point for TST was 5 mm in HIV-infected individuals and 15 mm in HIV-uninfected.

*McNemar’s test. In an intention to diagnose principle, QFT indeterminate results were included as negative.

^†^Including 97 participants (≤5 years of age) with unknown HIV status.

^‡^Excluding 162 with missing QFT. 15/43 QFT indeterminate results were C-Tb positives.

^§^17 with missing QFT. 15/68 QFT indeterminate results were C-Tb positives.

Excluding the 44 participants who were empirically treated for TB from the 920 included in the non-TB group reduced the C-Tb test positive rate by only 2% points (data not shown).

### CD4

**Fig 2:**
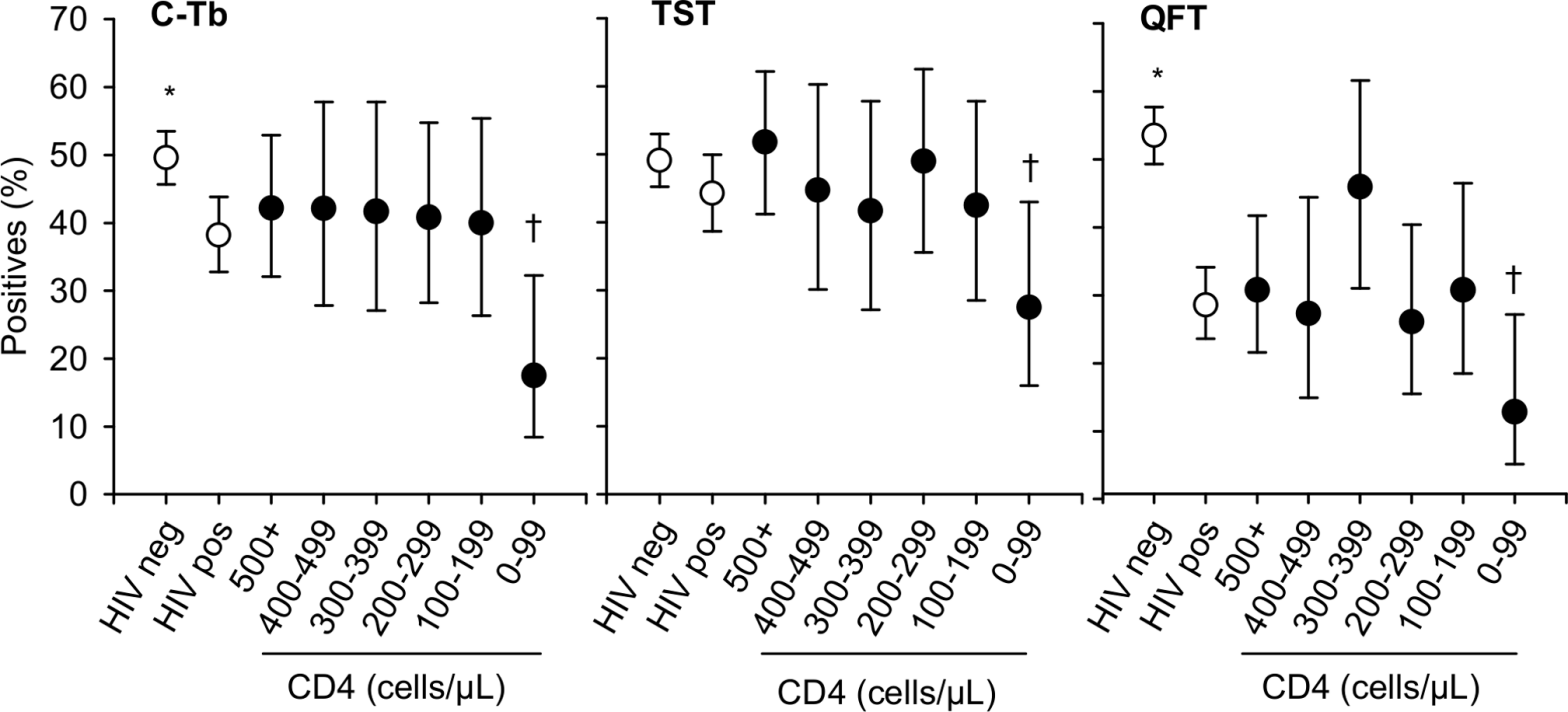
Test positivity rate according to HIV status and CD4 count. The data shown excludes participants with unknown HIV status and healthy control group. Error bars indicate 95% CI. Cut-point for TST was 5 mm in HIV-infected and 15 mm in others. C-Tb: N = 921 (625 HIV-uninfected, 296 HIV-infected). TST: N = 923 (627 HIV-uninfected, 296 HIV-infected). QFT: N = 818 (126 indeterminate, 538 HIV-uninfected, 280 HIV-infected). Data specific to the active and non-TB groups are shown in S2–S4 Tables. *HIV-uninfected versus HIV-infected p<0.05. ^†^CD4 counts ≥100 cells/μL versus counts <100 cells/μL in HIV-infected; p<0.05.

All three tests showed reduced test-positivity rates in HIV infected participants with CD4 counts <100 cells/μL (Fig 2). This was observed in both those diagnosed with active TB and those without active TB (S5 and S6 Tables). For QFT, the rate of indeterminate results reached a maximum of 65% (17/26) at CD4 counts <100 cells/μL.

### Children

In children <5 years old TST results are used to guide treatment for active TB. In the present trial tuberculosis was diagnosed in 12/148 (8%) symptomatic children <5 years old, two of them were confirmed microbiologically and ten diagnosed according to symptoms or signs of TB (S7 Table). The two participants diagnosed with active TB both showed indurations ≥15 mm with both skin agents, the youngest at four months of age. Both were close contacts with a PTB case and presented with cough >2 weeks and drenching night sweats. Those diagnosed according to clinical symptoms all had TST indurations ≥14 mm, and all but three tested positive with C-Tb. Of note, 12 children presenting with similar symptoms and positive C-Tb results were not diagnosed with TB (S6 Table). Four of those had no TST induration.

In the asymptomatic group of children with PTB contacts, 38% (33/87) tested positive with C-Tb or TST, 23 being positive with the two tests and five positive with either (p = 0.7518, concordance 89%, Table 4).

**Table 4:** Test positive results of C-Tb versus TST in children <5 years.

Group	Age (years)	N	C-Tb pos. (%)	TST pos. (%)	p-value*
**Asymptomatic contacts**	<5	87	32.2 (23.3–42.6)	32.2 (23.3–42.6)	0.7518
	0–1	30	23.3 (11.5–41.2)	23.3 (11.5–41.2)	0.6171
	2–4	57	36.8 (25.5–49.9)	36.8 (25.5–49.9)	0.6831
**Symptomatic** **active**^†^ **and non-TB**	<5	148	14.2 (9.4–20.8)	17.6 (12.2–24.6)	0.3320
	0–1	69	10.1 (4.7–19.8)	7.3 (2.8–16.2)	0.7237
	2–4	79	17.7 (10.7–27.7)	26.6 (18.0–37.3)	0.0455

Data are presented as % (95% CI). Cut-point for TST was 15 mm.

*McNemar’s test.

^†^Active TB was diagnosed in two, one in each age group 0–1 and 2–4 years.

The youngest participant testing positive with C-Tb had an induration of 11 mm at 71 days of age, and the youngest responder to TST had an induration of 9 mm at 53 days of age.

**Fig 3:**
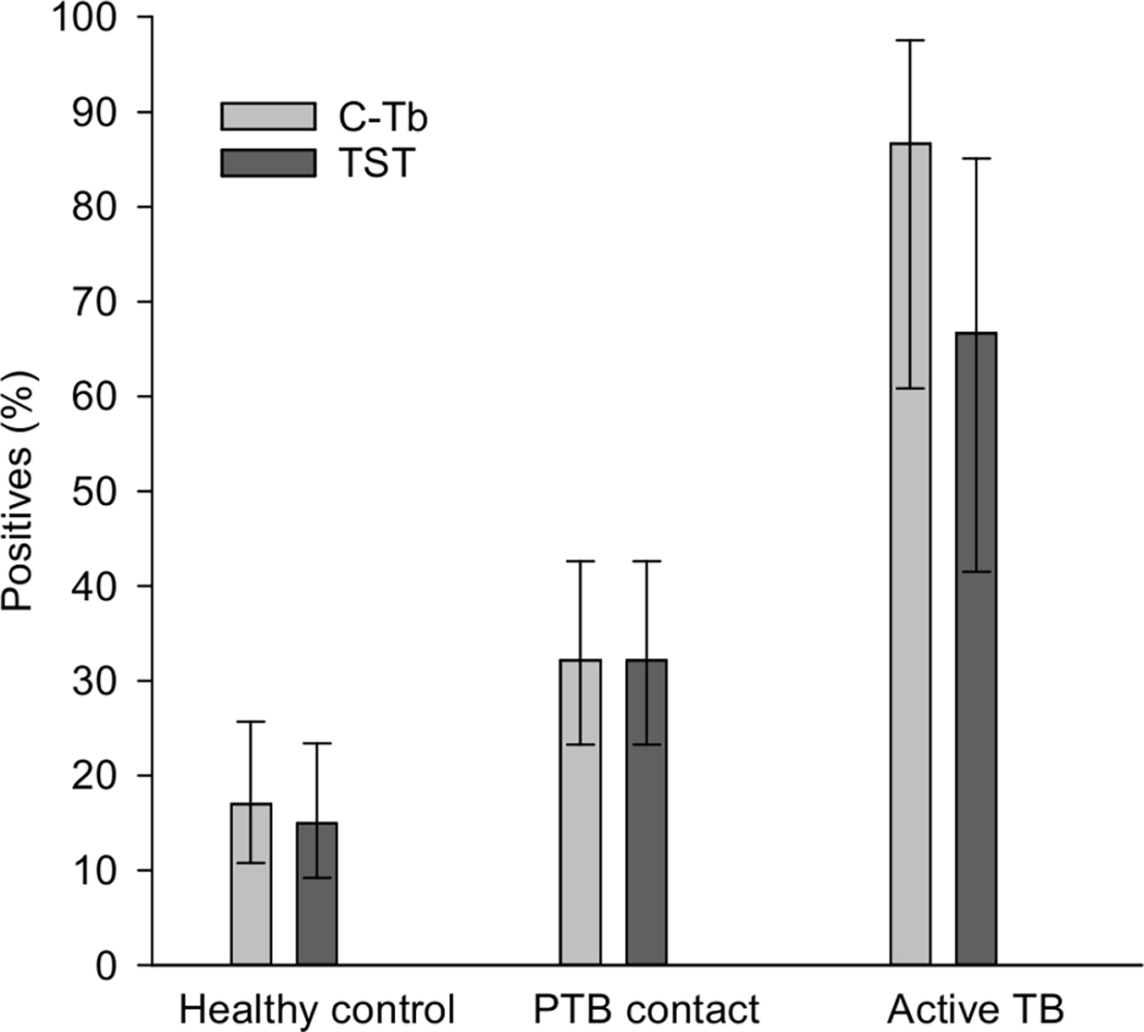
Test positivity rates in different subgroups of children comparing TST and C-Tb along an exposure gradient. Healthy control group (5–11 years, n = 100), asymptomatic PTB contacts <5 years (n = 87) and active TB cases <18 years (n = 15, no HIV-infected). Error bars indicate 95% CI.

C-Tb showed an increasing test positivity rate with age (S8 Table) and with exposure (Fig 3). Thus, 17% (17/100) in the healthy control group tested positive with C-Tb, increasing to 32% (28/87) of the PTB contacts and 87% (13/15) in those with active TB (p<0.0001). Similar results were found for TST (S9 and S10 Tables).

### Induration size

To explore the impact of age and HIV status on skin test performance we compared the induration sizes of C-Tb and TST responders (defined as skin test induration ≥1 mm) and the number of non-responders (bimodal distribution). C-Tb showed a minor but significantly decreased induration from median 21 to 19 mm in HIV-infected participants aged 5–65 years (p = 0.0148), whereas TST showed a median of 20 mm irrespective of HIV status (S1 Fig). The number of non-responding active and non-TB participants (5–65 years) increased significantly from 43% (238/550) in HIV-uninfected to 57% (169/296) in HIV-infected for C-Tb, and from 32% (178/551) to 51% (152/296) for TST (p<0.005). Also age had little influence on the medians (S2 Fig). However, symptomatic children aged below two showed a significantly reduced median of 4 mm (C-Tb, p<0.0001, One Way ANOVA) and 10 mm (TST, p<0.0001) respectively, indicating that indurations were influenced by symptoms in the youngest children (S2 Fig and S10 Table). Further, the positivity rates for both agents were significantly lower in symptomatic children <5 years old compared to asymptomatic children (Table 4, χ^2^, p<0.05). Of note, the median C-Tb induration among TB symptomatic responders was 20 mm irrespective of race (394 of Black African ancestry; 111 mixed European/African, p = 0.4520). TST showed a median of 20 mm in participants of Black African ancestry (n = 483), and was significantly reduced to 17 mm in participants of mixed ancestry (n = 128, p = 0.0335), which was associated with the higher BCG vaccination coverage of 85% in the mixed race group compared to 70% in the group with Black African ancestry.

### Safety

No serious AEs related to either of the skin tests were observed by the investigators. More than 95% were of mild-moderate intensity. A quarter of the participants experienced at least one ISR from both agents (Table 5).

**Table 5:** Injection site reactions in the full analysis set.

	C-Tb (N = 1188)	TST (N = 1190)
**All reactions**	282 (23.7)	290 (24.4)
**Pruritus**	210 (17.7)	221 (18.6)
**Pain**	90 (7.6)	81 (6.8)
**Rash**	58 (4.9)	63 (5.3)
**Vesicles**	24 (2.0)	24 (2.0)
Induration*	15 (1.3)	8 (0.7)
**Swelling**	5 (0.4)	4 (0.3)
**Erythema**^†^	3 (0.3)	3 (0.3)

Observations are presented as n (%). N = total number of injections.

*Induration ≥50 mm.

^†^Erythema ≥80 mm.

C-Tb showed similar types and frequencies of ISRs as TST, of which pruritus, pain, and rash were the most common. Injection site reactions appeared most frequently among responders, indicating that the reactions were inevitable parts of the delayed-type hypersensitivity reaction. Hence, adults showed more ISRs than children, and HIV-uninfected more than HIV-infected (not shown). No haematoma were reported with C-Tb.

A total of 338 (28%) participants reported 550 systemic AEs, of which headache (107 [9.0%]) and pyrexia (29 [2.4%]) were the most common.

## Discussion

In this phase III trial we found that C-Tb had similar sensitivity compared to the TST and QFT, both in children and HIV co-infected persons with active TB. C-Tb also had similar test-positivity compared to QFT and TST in those with presumed LTBI (i.e. non-TB). These results support the cut-point of C-Tb determined in adults [12,13] may also be applied in children and in HIV-infected individuals. Separate dose-finding and sensitivity trials from the outset (initial phase 1, 2 and 3 trials) would not have been feasible in infants and children due to the paucibacillary nature of TB, their inability to expectorate, and frequent occurrence of extrapulmonary TB [18]. Hence, the need for the current trial in these vulnerable, and often neglected subgroups of patients. Indeed, one of the strategies strongly recommended by WHO to eliminate TB worldwide is systematic testing and treatment of LTBI in people living with HIV, in children, and now also in adolescents and adults, who are in close contact with PTB cases [3,19]. The major potential advantages of C-Tb in endemic countries would be: (i) lack of the need for laboratory facilities equipped with plate readers; these are in short supply and where they exist there is no additional capacity; (ii) lack of the need for additional technicians which are already in short supply; (iii) lack of the need for phlebotomy which is a major hurdle in children; (iv) lack of the need for a transport infrastructure which is already overburdened; (iv) easier test operation by existing health care workers; (v) substantially lower cost (although not yet finalised the tiered pricing for C-Tb will be competitive with TST; current cost of an IGRA to the privately paying end user in South Africa is ~US$ 77 and ~ US$ 10 for the TST [20]).

In keeping with earlier preliminary findings [13], all three tests showed reduced sensitivities in those with CD4 T cell counts below 100 cells/μL, reflecting that the tests relies on competent immune function. The cut-points of TST (5 mm in HIV-infected, 15 mm in others) and C-Tb (5 mm applied in the present trial) were validated in earlier trials [12,13] and in a separate trial that will report a sensitivity of C-Tb similar to TST and QFT (Aggerbeck, in preparation). In the present trial, the positivity rate of C-Tb was similar to TST and QFT except in HIV-infected non-TB participants where C-Tb appeared more sensitive than QFT (but less sensitive than TST) due to a high number of indeterminate results. The reasons for this observation remain unclear but may be related to incubation time with antigen (higher with skin tests) and antigen diversity and load (higher with TST). TST showed the same median induration among HIV-infected and HIV-uninfected responders (induration ≥1 mm), indicating that a reduced positivity rate among HIV-infected persons is driven by the increased number of non-responders. These non-responders would have remained test-negative even if a 5 mm cut-point was applied, however the specificity (63%) would be reduced, as reported previously [8,12,21].

One third of the asymptomatic childhood contacts of PTB aged <5 years old tested positive with C-Tb and TST; it is likely that these children may benefit from LTBI treatment [3]. QFT was not done in this age group and in accordance with WHO recommendations and the reluctance by this group to accept phlebotomy [22–24]. Among symptomatic children <5 years old the test-positivity rate of C-Tb and TST was significantly reduced compared to asymptomatic children. Furthermore, the median induration size was reduced, especially in children aged below two. This may be due to the immune-suppressive effect of TB itself, highlighting the need to carefully interpret a negative test result. Consequently, although not investigated in the present trial, in some cases, a repeat test may be considered after 2–3 months on treatment for active TB when the severe illness has resolved [15]. We have previously shown that a repeat test with C-Tb may be feasible [25].

There are several limitations to our trial, including the inherent difficulties in diagnosing active TB in children resulting in misclassification bias (a limitation in all diagnostic trials in children). Indeed, among all paediatric cases reported from 2000 to 2009 in the EU region, only 17% were culture-confirmed [26]. In this trial, QFT had a high percentage (14%) of indeterminate results, resulting in a low test positivity rate in HIV-infected persons. However, a pooled rate of 15% indeterminate results have been reported from high burden countries [27], and high indeterminate rates have been associated with low CD4 counts [28]. Moreover, the test was done by an ISO 15189 certified laboratory, and with a stringent quality assurance system. For example, date and time when samples were collected and received at the laboratory were recorded in addition to incubation conditions as part of the laboratory standard operating procedures. This revealed five cases incubated at an elevated temperature (these results were recorded as missing), and five samples were delayed during transport. It is well known that delays in incubation or a problem in one of the other steps may contribute to the number of indeterminate results [11]. Of note, both skin tests classified one third of the 125 QFT indeterminate results as *M*.*tb* infected cases, indicating that simple skin tests may be more suitable than laboratory-dependent tests in resource constrained settings. High QFT sensitivity has been reported in low TB endemic countries [29,30].

We measured sensitivity in active TB. However, as there is no reference standard to diagnose LTBI, sensitivity can only be measured in active TB, and only comparative descriptive statistics (comparing positivity of C-Tb, QFT, and TST) provided in those with non-TB. Alternatively, in the absence of a gold standard, the sensitivity and the specificity of any two tests may be *estimated* from groups with different prevalence using a latent class model [31,32], although such estimates may be biased if the tests have different sensitivities in the various subgroups as reported here. Moreover, such an approach is still an approximation and can never compensate fully for lack of a gold standard. Although we could not detect any difference in test positivity rates between C-Tb and QFT (excluding indeterminate results), it is possible that small but significant differences could have been missed given the limited sample size. However, the study was primarily powered to detect rare adverse events and not small but potentially significant inter-group differences. Finally, if it is hypothesized that C-Tb (containing RD-1 antigens only) is a more specific test than TST, then positivity rates of C-Tb in the non-TB group should be lower than TST (which was not the case). We believe this is due to the lack of a BCG confounding effect when given at birth. Indeed, a systematic review involving over 240 000 participants enrolled in 24 trials found that the effect of BCG vaccination on TST, when given at birth (the South African practice), was minimal at the 15 mm cut-point [8,33,34].

There was significant racial disparity in the healthy control group (none were Black African); this inadvertently arose due to the population distribution of the sites where the healthy controls were recruited. However, in all the children (excluding the healthy controls) the C-Tb induration size was similar in those of Black African and mixed ancestry. A higher number of responders (but lower median TST induration) was observed in participants of mixed race, which could reflect the higher BCG vaccination coverage in participants of mixed race (85%) compared to Black Africans (70%) [6].

In conclusion, C-Tb has a similar sensitivity compared to TST and QFT in adults and children with active TB or presumed LTBI using a universal 5 mm cut-point. In HIV-infected persons C-Tb appeared more sensitive than QFT. These informative data could be valuable for clinical practice in the future, suggesting that C-Tb may be a useful tool to guide LTBI-specific treatment as outlined in the WHO End TB Strategy [3,16].

## Supporting information

S1 File Methods. (DOCX)

S1 Fig Median induration in symptomatic participants (5–65 years) according to HIV status. Line within boxes are medians of responders (induration ≥1 mm), boundaries of boxes represents 25^th^ to 75^th^ percentiles, and error bar 90^th^ percentiles. *p<0.05. C-Tb: HIV-uninfected: n = 312, HIV-infected: n = 127. TST: HIV-uninfected: n = 373, HIV-infected: n = 144.
(TIF)

S2 Fig Median induration according to age, symptoms, and HIV. Ctr is control group (5–11 years). Line within boxes are medians of responders (induration ≥1 mm), boundaries of boxes represents 25^th^ to 75^th^ percentiles, and error bar 90^th^ percentiles. *: p<0.05. C-Tb: Asymptomatic PTB contacts <5 years: n = 30; Control 5–11 years: n = 17. Symptomatic HIV-uninfected: <5 years: n = 37; 5–11 years: n = 63; 12–65 years: n = 249. HIV-infected: n = 127. TST: Asymptomatic PTB contacts <5 years: n = 30; Control 5–11 years: n = 28. Symptomatic HIV-uninfected: <5 years: n = 56; 5–11 years: n = 67; 12–65 years: n = 306. HIV-infected: n = 144
(TIF)

S1 Table Symptoms and signs of TB. Data are presented as n (%). *Sweat that requires the patient to change clothes. ^†^Failure to gain weight, and loss of appetite (only children).
(DOCX)

S2 Table Result of C-Tb versus TST and QFT in microbiologically confirmed cases of active TB according to HIV status. Left: C-Tb versus TST. Middle: C-Tb versus QFT. Right: TST versus QFT. *McNemar’s test. Cut-point for TST was 5 mm for HIV-infected and 15 mm for others. In an intention to diagnose principle, QFT indeterminate results were regarded as negative (arrows). ^†^Excluding 3 with missing QFT. ^‡^Excluding 2 with missing QFT.
(DOCX)

S3 Table Results of C-Tb versus TST and QFT in non-TB participants according to HIV status. Left: C-Tb versus TST. Middle: C-Tb versus QFT. Right: TST versus QFT. *McNemar’s test. Cut-point for TST was 5 mm for HIV-infected and 15 mm for others. In an intention to diagnose principle, QFT indeterminate results were regarded as negative (arrows). ^†^Excluding 162 (161) with missing QFT. ^‡^Excluding 17 with missing QFT.
(DOCX)

S4 Table Results of C-Tb versus TST and QFT in active and non-TB participants according to HIV status. Left: C-Tb versus TST. Middle: C-Tb versus QFT. Right: TST versus QFT. *McNemar’s test. Cut-point for TST was 5 mm for HIV-infected and 15 mm for others. In an intention to diagnose principle, QFT indeterminate results were regarded as negative (arrows). ^†^Excluding 165 (166) with missing QFT. ^‡^Excluding 19 with missing QFT.
(DOCX)

S5 Table Sensitivity in HIV co-infected active TB cases according to CD4 count. Data are presented as % (95% CI). Cut-point for TST was 5 mm. *Fishers Exact test. ^†^Based on those tested including 5 with indeterminate results. ^‡^2 missing QFT but including 4 with indeterminate results.
(DOCX)

S6 Table Test positive result of C-Tb, TST, and QFT in non-TB, HIV-infected participants according to CD4 count. Data are presented as % (95% CI). Cut-point for TST was 5 mm. *Excluding 10 with missing CD4 count. ^†^χ^2^ test. ^‡^Excluding 2 with missing QFT test, but including 17 with indeterminate results. ^§^Excluding 13 with missing QFT test, but including 48 with indeterminate outcome.
(DOCX)

S7 Table Symptoms and TB in children aged below 5. The table shows all children below five with microbiologically confirmed TB, those diagnosed according to clinical symptoms, and those with a positive C-Tb result but no TB diagnosis.
(DOCX)

S8 Table Results of C-Tb, TST, and QFT in 704 active and non-TB participants according to age. Data are presented as n (%). Cut-points defining positive results were 5 mm (C-Tb) and 15 mm (TST). *Excluding 299 HIV-infected participants due to an uneven distribution in the various age groups. ^†^Median (IQR) among responders with induration ≥1 mm.
(DOCX)

S9 Table Results of C-Tb versus TST and QFT in healthy control group aged 5–11 years. *McNemar’s test. Cut-point for TST was 15 mm. In an intention to diagnose principle, QFT indeterminate results were regarded as negative (arrows). ^†^2 missing QFT.
(DOCX)

S10 Table Results of C-Tb versus TST in asymptomatic and symptomatic children <5 years. *McNemar’s test. Cut-point for TST was 15 mm. ^†^Including two with active TB.
(DOCX)
