# Supplementary material for: C-Tb skin test to diagnose Mycobacterium tuberculosis infection in children and HIV-infected adults: A phase 3 trial
Source: PLoS One. 2018 Sep 24;13(9):e0204554. doi: 10.1371/journal.pone.0204554 (PMC6152999; doi:10.1371/journal.pone.0204554)
Supplement: S1 File — (DOCX) [file pone.0204554.s001.docx]

**Trial design and participants**

The trial was a randomized, double-blind split-body design conducted at eight sites in South Africa: In Cape Town at Karl Bremer Hospital (Tiervlei Trial Centre, Delft Community Health Care Centre (TASK), and Groote Schuur Hospital(Langa Clinic), Paarl (Be Part Yoluntu Centre), Pretoria (Setshaba Research Centre and Synexus Stanza Bopape Clinic), Port Elizabeth (Primecure Medicentre), and in Benoni (Lakeview Hospital). The trial was approved by Pharma-Ethics (No. 12024740), by the University of Cape Town Human Research Ethics Committee (No. 222/2012), by the Medicines Control Council (No. 20120120). The trial was part of an agreed Paediatric Investigation Plan, which is in accordance with EU regulations.

**Randomization and masking**

Identical appearing vials of C-Tb and PPD RT 23 filled in ten dose vials were labelled left or right and randomized in blocks of ten (five left and five right of each agent) and kept in sequentially numbered cardboard boxes with two vials in each (one vial of each agent). An independent statistician programmed the random permutations in SAS. Sealed emergency envelopes for each participant, prepared by the quality assurance department at SSI by personnel not involved in the trial, made it possible to reveal the actual allocation of the test agents without unblinding the whole trial.

**Procedures**HIV-infection was diagnosed by two alternative positive rapid tests (OraQuick^®^ and First Response^TM^ HIV1-2.0 Card Test) or one positive rapid test and a confirmatory ELISA (Enzygnost^®^ Anti-HIV-1/2 Plus). In the age group below five years of age, the HIV test taken right after birth was used. CD4 count was determined on HIV-infected participants.

Blood samples for QuantiFERON^®^-TB Gold In Tube (Qiagen, Hilden, Germany) were collected from participants aged five years and older prior to administration skin test agents to avoid a booster response. QFT was done by PathCare (Goodwood, South Africa). Results were reported as positive, negative or indeterminate according to manufacturer’s instruction based on a cut-point of 0.35 IU/mL. Test results were indeterminate if the positive control was <0.5 IU/mL or the negative control >8.0 IU/mL.

C-Tb was administered in a dose of 0.1 µg and PPD RT 23 in a dose of 2 TU with injections of 0.1 mL in separate arms according to the Mantoux technique. Test positivity were defined by indurations ≥5 mm for C-Tb, and 5 or 15 mm for TST (5 mm if HIV-infected) measured 48-72 h after intradermal injection transversely to the long axis of the forearm using the ‘Ballpoint method’. In an attempt to exclude uninfected, indurations ≥1 mm defined a responder.

The trial included four visits: Screening visit (≤ 28 days before testing), randomization visit at Day 0 which included taking blood samples for QFT analysis and performing the two skin tests, follow-up visit after 48-72 h where reading of induration responses and safety assessments were recorded, and combined follow-up and trial termination visit at Day 28 where induration responses and AEs were recorded. In addition, safety blood tests were taken in all participants five years and older and medical examinations performed.

**Statistical analysis**

Given the lack of a microbiological definition of LTBI, sensitivity was determined in participants with active TB. In those without active TB (non-TB), C-Tb test-positivity rates were compared to QFT and TST. Differences in number of test positives were assessed by McNemar’s test for marginal heterogeneity, and in unpaired analyses with Fisher’s exact test or χ^2^ test. Agreement between tests was assessed with Cohen’s κ coefficient. The trial was optimally sized to detect rare AEs with an upper 95% limit corresponding to a total of 3000 participants. The aim was to include at least 60 participants in each age group (0-1, 2-4, 5-11, 12-17, 18-39, and 40-65), (500 children plus 100 in the control group). We had planned to enroll 300 people living with HIV, 30% of the adult population had to be below 40 years old, and 30% above 40 years old. Each gender had to constitute at least 40% of the total recruited. Statistical analysis and graphs were prepared in SAS 9.3 and SigmaPlot 13.
