# Supplementary figures and images for: C-Tb skin test to diagnose Mycobacterium tuberculosis infection in children and HIV-infected adults: A phase 3 trial
Source: PLoS One. 2018 Sep 24;13(9):e0204554. doi: 10.1371/journal.pone.0204554 (PMC6152999; doi:10.1371/journal.pone.0204554)

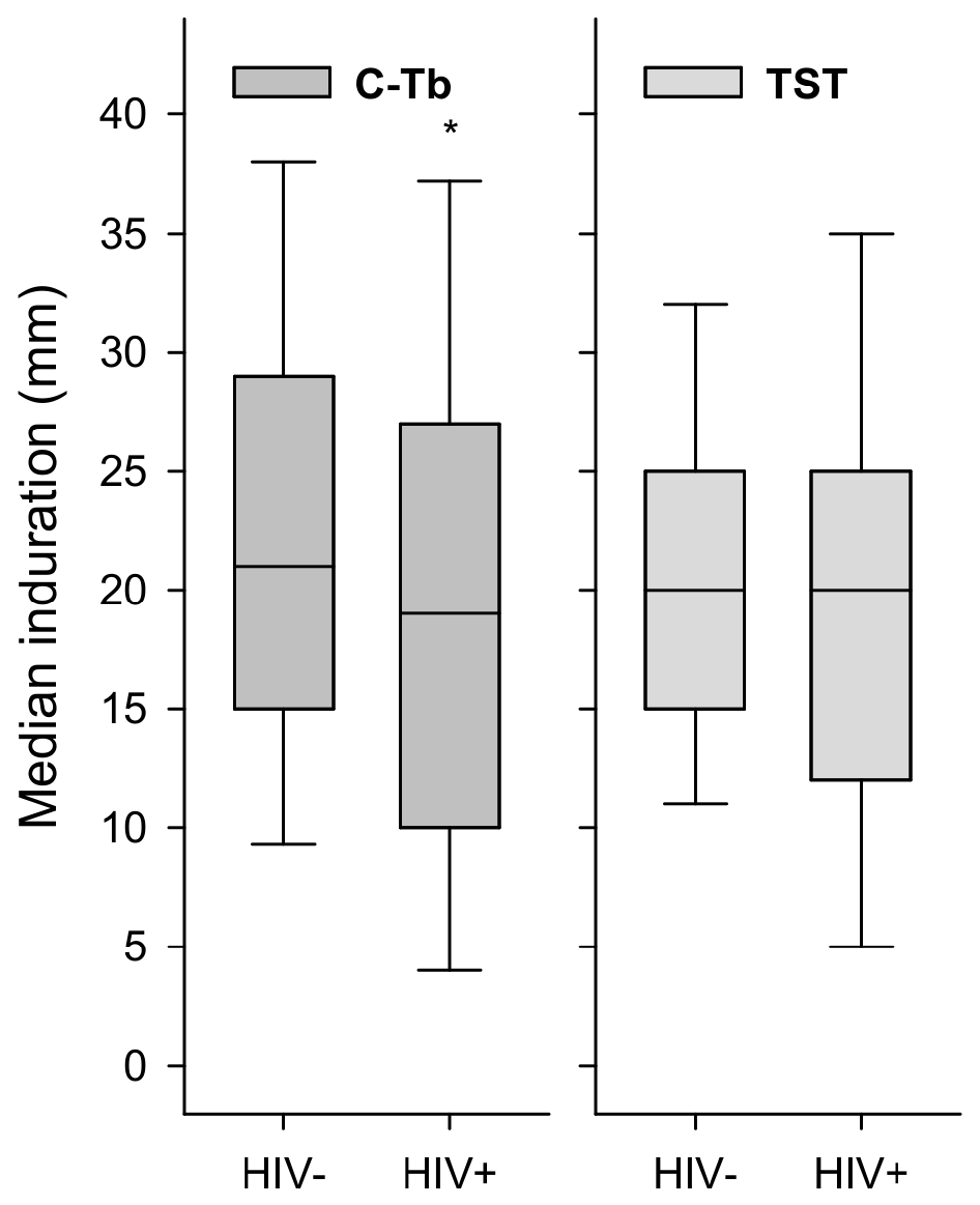

Supplement: S1 Fig — Line within boxes are medians of responders (induration ≥1 mm), boundaries of boxes represents 25th to 75th percentiles, and error bar 90th percentiles. *p<0.05. C-Tb: HIV-uninfected: n = 312, HIV-infected: n = 127. TST: HIV-uninfected: n = 373, HIV-infected: n = 144. (TIF) [file pone.0204554.s002.tif]

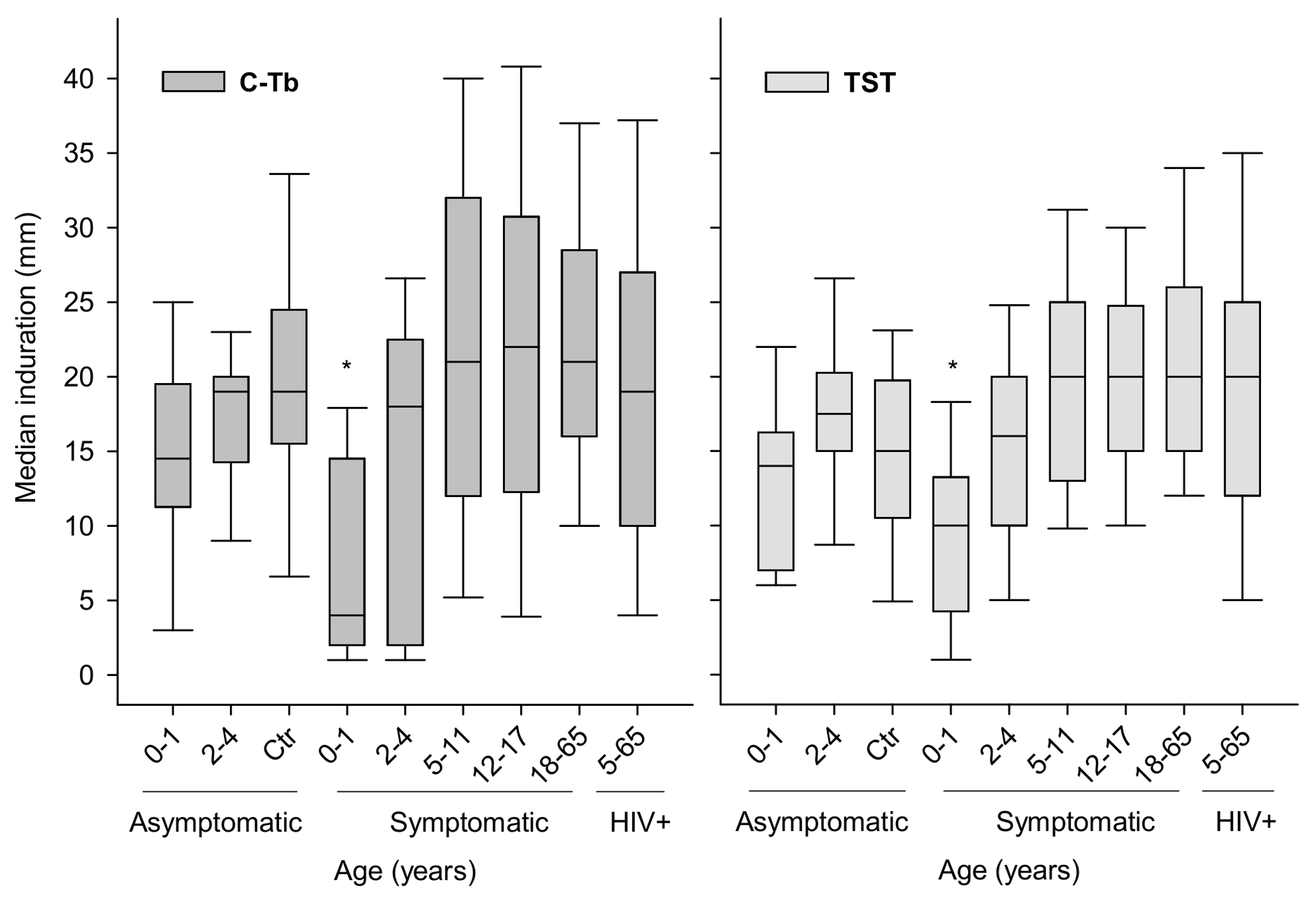

Supplement: S2 Fig — Ctr is control group (5–11 years). Line within boxes are medians of responders (induration ≥1 mm), boundaries of boxes represents 25th to 75th percentiles, and error bar 90th percentiles. *: p<0.05. C-Tb: Asymptomatic PTB contacts <5 years: n = 30; Control 5–11 years: n = 17. Symptomatic HIV-uninfected: <5 years: n = 37; 5–11 years: n = 63; 12–65 years: n = 249. HIV-infected: n = 127. TST: Asymptomatic PTB contacts <5 years: n = 30; Control 5–11 years: n = 28. Symptomatic HIV-uninfected: <5 years: n = 56; 5–11 years: n = 67; 12–65 years: n = 306. HIV-infected: n = 144 (TIF) [file pone.0204554.s003.tif]
