# Supplementary material for: C-Tb skin test to diagnose Mycobacterium tuberculosis infection in children and HIV-infected adults: A phase 3 trial
Source: PLoS One. 2018 Sep 24;13(9):e0204554. doi: 10.1371/journal.pone.0204554 (PMC6152999; doi:10.1371/journal.pone.0204554)
Supplement: S1 Table — Data are presented as n (%). *Sweat that requires the patient to change clothes. †Failure to gain weight, and loss of appetite (only children). (DOCX) [file pone.0204554.s004.docx]

|  | **All** | **0-4 years** | **5-11 years** | **12-17 years** | **18-39 years** | **40-65 years** |
| --- | --- | --- | --- | --- | --- | --- |
| **N** | 1003 | 149 | 170 | 96 | 311 | 277 |
| **Cough >2 weeks** | 938 (93.5) | 137 (91.9) | 158 (92.9) | 87 (90.6) | 292 (93.9) | 264 (95.3) |
| **Drenching night sweats^*^** | 724 (72.2) | 93 (62.4) | 113 (66.5) | 51 (53.1) | 244 (78.5) | 223 (80.5) |
| **Weight loss** | 545 (54.3) | 48 (32.2) | 78 (45.9) | 37 (38.5) | 206 (66.2) | 176 (63.5) |
| **Feverish >2 weeks** | 488 (48.7) | 42 (28.2) | 104 (61.2) | 35 (36.5) | 173 (55.6) | 134 (48.4) |
| **Enlarged cervical lymphadenopathy** | 264 (26.3) | 60 (40.3) | 79 (46.5) | 21 (21.9) | 55 (17.7) | 49 (17.7) |
| **Failure to thrive^†^** | 14 (3.4) | 10 | 3 | 1 | N.A. | N.A. |
