# Supplementary material for: C-Tb skin test to diagnose Mycobacterium tuberculosis infection in children and HIV-infected adults: A phase 3 trial
Source: PLoS One. 2018 Sep 24;13(9):e0204554. doi: 10.1371/journal.pone.0204554 (PMC6152999; doi:10.1371/journal.pone.0204554)
Supplement: S2 Table — Left: C-Tb versus TST. Middle: C-Tb versus QFT. Right: TST versus QFT. *McNemar’s test. Cut-point for TST was 5 mm for HIV-infected and 15 mm for others. In an intention to diagnose principle, QFT indeterminate results were regarded as negative (arrows). †Excluding 3 with missing QFT. ‡Excluding 2 with missing QFT. (DOCX) [file pone.0204554.s005.docx]

| **All)** |  | **C-Tb** | |  |  | **All)** |  | **C-Tb** | |  |  | **All)** |  | **TST** | |  |
| --- | --- | --- | --- | --- | --- | --- | --- | --- | --- | --- | --- | --- | --- | --- | --- | --- |
|  |  | **Pos** | **Neg** | **∑** |  |  |  | **Pos** | **Neg** | **∑** |  |  |  | **Pos** | **Neg** | **∑** |
| **TST** | **Pos** | 47 | 9 | 56 |  | **QFT** | **Pos** | 35 | 6 | 41 |  | **QFT** | **Pos** | 29 | 12 | 41 |
|  |  |  |  |  |  |  | **Ind** | 10↓ | 4↓ | 14 |  |  | **Ind** | 11↓ | 3↓ | 14 |
|  | **Neg** | 7 | 12 | 19 |  |  | **Neg** | 4 | 11 | 15 |  |  | **Neg** | 11 | 4 | 15 |
|  | **∑** | 54 | 21 | 75 |  |  | **∑** | 49 | 21 | 70 |  |  | **∑** | 51 | 19 | 70 |
|  | p^*^=0.8026;  κ=0.46 (0.23-0.68)  Concordance=78.7% | | | |  |  | p^*^=0.1175;  κ=0.39 (0.17-0.60)  Concordance=71.4% | | | |  |  | p^*^=0.1227;  κ=-0.05 (-0.27-0.17)  Concordance=51.4% | | | |

| **HIV-ve)** | | **C-Tb** | |  |  | **HIV-ve)** | | **C-Tb** | |  |  | **HIV-ve)** | | **TST** | |  |
| --- | --- | --- | --- | --- | --- | --- | --- | --- | --- | --- | --- | --- | --- | --- | --- | --- |
|  |  | **Pos** | **Neg** | **∑** |  |  |  | **Pos** | **Neg** | **∑** |  |  |  | **Pos** | **Neg** | **∑** |
| **TST** | **Pos** | 25 | 5 | 30 |  | **QFT^†^** | **Pos** | 22 | 4 | 26 |  | **QFT** | **Pos** | 17 | 9 | 26 |
|  |  |  |  |  |  |  | **Ind** | 5↓ | 0↓ | 5 |  |  | **Ind** | 5↓ | 0 | 5 |
|  | **Neg** | 6 | 5 | 11 |  |  | **Neg** | 1 | 6 | 7 |  |  | **Neg** | 5 | 2 | 7 |
|  | **∑** | 31 | 10 | 41 |  |  | **∑** | 28 | 10 | 38^†^ |  |  | **∑** | 27 | 11 | 38^†^ |
|  | p^*^=1.0000;  κ=0.30 (-0.03-0.62)  Concordance=73.2% | | | |  |  | p^*^=0.7518;  κ=0.36 (0.04-0.68)  Concordance=73.7% | | | |  |  | p^*^=1.0000;  κ=-0.18 (-0.47-0.10)  Concordance=50.0% | | | |

| **HIV+ve)** | | **C-Tb** | |  |  | **HIV+ve)** | | **C-Tb** | |  |  | **HIV+ve)** | | **TST** | |  |
| --- | --- | --- | --- | --- | --- | --- | --- | --- | --- | --- | --- | --- | --- | --- | --- | --- |
|  |  | **Pos** | **Neg** | **∑** |  |  |  | **Pos** | **Neg** | **∑** |  |  |  | **Pos** | **Neg** | **∑** |
| **TST** | **Pos** | 22 | 4 | 26 |  | **QFT^‡^** | **Pos** | 13 | 2 | 15 |  | **QFT** | **Pos** | 12 | 3 | 15 |
|  |  |  |  |  |  |  | **Ind** | 5↓ | 4↓ | 9 |  |  | **Ind** | 6↓ | 3↓ | 9 |
|  | **Neg** | 1 | 7 | 8 |  |  | **Neg** | 3 | 5 | 8 |  |  | **Neg** | 6 | 2 | 8 |
|  | **∑** | 23 | 11 | 34 |  |  | **∑** | 21 | 11 | 32^‡^ |  |  | **∑** | 24 | 8 | 32^‡^ |
|  | p^*^=0.3711;  κ=0.64 (0.36-0.92)  Concordance=85.3% | | | |  |  | p^*^=0.1138;  κ=0.39 (0.09-0.68)  Concordance=68.8% | | | |  |  | p^*^=0.0389;  κ=0.09 (-0.20-0.38)  Concordance=53.1% | | | |
