# Supplementary material for: C-Tb skin test to diagnose Mycobacterium tuberculosis infection in children and HIV-infected adults: A phase 3 trial
Source: PLoS One. 2018 Sep 24;13(9):e0204554. doi: 10.1371/journal.pone.0204554 (PMC6152999; doi:10.1371/journal.pone.0204554)
Supplement: S3 Table — Left: C-Tb versus TST. Middle: C-Tb versus QFT. Right: TST versus QFT. *McNemar’s test. Cut-point for TST was 5 mm for HIV-infected and 15 mm for others. In an intention to diagnose principle, QFT indeterminate results were regarded as negative (arrows). †Excluding 162 (161) with missing QFT. ‡Excluding 17 with missing QFT. (DOCX) [file pone.0204554.s006.docx]

| **All)** |  | **C-Tb** | |  |  | **All)** |  | **C-Tb** | |  |  | **All)** |  | **TST** | |  |
| --- | --- | --- | --- | --- | --- | --- | --- | --- | --- | --- | --- | --- | --- | --- | --- | --- |
|  |  | **Pos** | **Neg** | **∑** |  |  |  | **Pos** | **Neg** | **∑** |  |  |  | **Pos** | **Neg** | **∑** |
| **TST** | **Pos** | 306 | 85 | 391 |  | **QFT** | **Pos** | 256 | 68 | 324 |  | **QFT** | **Pos** | 237 | 88 | 325 |
|  |  |  |  |  |  |  | **Ind** | 30↓ | 81↓ | 111 |  |  | **Ind** | 32↓ | 79↓ | 111 |
|  | **Neg** | 71 | 458 | 529 |  |  | **Neg** | 59 | 247 | 306 |  |  | **Neg** | 84 | 222 | 306 |
|  | **∑** | 377 | 543 | 920 |  |  | **∑** | 345 | 396 | 741 |  |  | **∑** | 353 | 389 | 742 |
|  | p^*^=0.2980;  κ=0.65 (0.60-0.70)  Concordance=83.0% | | | |  |  | p^*^=0.1105;  κ=0.57 (0.51-0.63)  Concordance=78.8% | | | |  |  | p^*^=0.0587;  κ=0.45 (0.38-0.51)  Concordance=72.5% | | | |

| **HIV-ve)** | | **C-Tb** | |  |  | **HIV-ve)** | | **C-Tb** | |  |  | **HIV-ve)** | | **TST** | |  |
| --- | --- | --- | --- | --- | --- | --- | --- | --- | --- | --- | --- | --- | --- | --- | --- | --- |
|  |  | **Pos** | **Neg** | **∑** |  |  |  | **Pos** | **Neg** | **∑** |  |  |  | **Pos** | **Neg** | **∑** |
| **TST** | **Pos** | 226 | 60 | 286 |  | **QFT^†^** | **Pos** | 210 | 49 | 259 |  | **QFT** | **Pos** | 191 | 69 | 260 |
|  |  |  |  |  |  |  | **Ind** | 15↓ | 28↓ | 43 |  |  | **Ind** | 11↓ | 32↓ | 43 |
|  | **Neg** | 61 | 311 | 372 |  |  | **Neg** | 39 | 155 | 194 |  |  | **Neg** | 56 | 138 | 194 |
|  | **∑** | 287 | 371 | 658 |  |  | **∑** | 264 | 232 | 496^†^ |  |  | **∑** | 258 | 239 | 497^(†)^ |
|  | p^*^=1.0000;  κ=0.63 (0.57-0.69)  Concordance=81.6% | | | |  |  | p^*^=0.6935;  κ=0.58 (0.51-0.66)  Concordance=79.2% | | | |  |  | p^*^=0.9317;  κ=0.45 (0.37-0.53)  Concordance=72.6% | | | |

| **HIV+ve)** | | **C-Tb** | |  |  | **HIV+ve)** | | **C-Tb** | |  |  | **HIV+ve)** | | **TST** | |  |
| --- | --- | --- | --- | --- | --- | --- | --- | --- | --- | --- | --- | --- | --- | --- | --- | --- |
|  |  | **Pos** | **Neg** | **∑** |  |  |  | **Pos** | **Neg** | **∑** |  |  |  | **Pos** | **Neg** | **∑** |
| **TST** | **Pos** | 80 | 25 | 105 |  | **QFT^‡^** | **Pos** | 46 | 19 | 65 |  | **QFT** | **Pos** | 46 | 19 | 65 |
|  |  |  |  |  |  |  | **Ind** | 15↓ | 53↓ | 68 |  |  | **Ind** | 21↓ | 47↓ | 68 |
|  | **Neg** | 10 | 147 | 157 |  |  | **Neg** | 20 | 92 | 112 |  |  | **Neg** | 28 | 84 | 112 |
|  | **∑** | 90 | 172 | 262 |  |  | **∑** | 81 | 164 | 245^‡^ |  |  | **∑** | 95 | 150 | 245^‡^ |
|  | p^*^=0.0180;  κ=0.72 (0.63-0.80)  Concordance=86.6% | | | |  |  | p^*^=0.0412;  κ=0.48 (0.36-0.59)  Concordance=78.0% | | | |  |  | p^*^=0.0004;  κ=0.38 (0.26-0.50)  Concordance=72.2% | | | |
