# Supplementary material for: C-Tb skin test to diagnose Mycobacterium tuberculosis infection in children and HIV-infected adults: A phase 3 trial
Source: PLoS One. 2018 Sep 24;13(9):e0204554. doi: 10.1371/journal.pone.0204554 (PMC6152999; doi:10.1371/journal.pone.0204554)
Supplement: S5 Table — Data are presented as % (95% CI). Cut-point for TST was 5 mm. *Fishers Exact test. †Based on those tested including 5 with indeterminate results. ‡2 missing QFT but including 4 with indeterminate results. (DOCX) [file pone.0204554.s008.docx]

|  | **CD4 (cells/µL)** | | **p^*^** |
| --- | --- | --- | --- |
|  | **<100** | **≥100** |  |
| **N** | 12 | 22 |  |
| **C-Tb pos.** | 41.7  (19.3-68.1) | 81.8  (60.9-93.3) | 0.0256 |
| **TST pos.** | 50.0  (25.4-74.6) | 90.9  (71.0-98.7) | 0.0127 |
| **QFT pos.** | 33.3^†^  (13.6-61.2) | 55.0^‡^  (34.2-74.2) | 0.2907 |
