# Supplementary material for: C-Tb skin test to diagnose Mycobacterium tuberculosis infection in children and HIV-infected adults: A phase 3 trial
Source: PLoS One. 2018 Sep 24;13(9):e0204554. doi: 10.1371/journal.pone.0204554 (PMC6152999; doi:10.1371/journal.pone.0204554)
Supplement: S6 Table — Data are presented as % (95% CI). Cut-point for TST was 5 mm. *Excluding 10 with missing CD4 count. †χ2 test. ‡Excluding 2 with missing QFT test, but including 17 with indeterminate results. §Excluding 13 with missing QFT test, but including 48 with indeterminate outcome. (DOCX) [file pone.0204554.s009.docx]

|  | **CD4 (cells/µL)** | | **p**^†^ |
| --- | --- | --- | --- |
|  | **<100** | **≥100** |  |
| **N^*^** | 28 | 224 |  |
| **C-Tb pos.** | 7.1  (0.9-23.7) | 37.5  (31.4-44.0) | 0.0029 |
| **TST pos.** | 17.9  (7.4-36.1)) | 42.9  (36.6-49.4) | 0.0193 |
| **QFT pos.** | 3.9^‡^  (0.0-20.5) | 29.9^§^  (24.1-36.4) | 0.0097 |
