# Supplementary material for: C-Tb skin test to diagnose Mycobacterium tuberculosis infection in children and HIV-infected adults: A phase 3 trial
Source: PLoS One. 2018 Sep 24;13(9):e0204554. doi: 10.1371/journal.pone.0204554 (PMC6152999; doi:10.1371/journal.pone.0204554)
Supplement: S7 Table — The table shows all children below five with microbiologically confirmed TB, those diagnosed according to clinical symptoms, and those with a positive C-Tb result but no TB diagnosis. (DOCX) [file pone.0204554.s010.docx]

| **TB diagnosed** | **Age (years)** | **Induration (mm)** | | **Cough** | **Night sweat** | **Lymphade-nopathy** | **Weight loss** | **Fever** | **Failure to thrive** |
| --- | --- | --- | --- | --- | --- | --- | --- | --- | --- |
|  |  | **C-Tb** | **TST** |  |  |  |  |  |  |
| **Confirmed** | 0 | 15 | 19 | ● | ● |  | ● |  |  |
|  | 2 | 25 | 26 | ● | ● | ● |  |  |  |
| **Clinical symptoms** | 1 | 17 | 14 | ● | ● | ● | ● |  |  |
|  | 2 | 27 | 20 | ● | ● | ● |  |  |  |
|  | 2 | 19 | 20 | ● |  | ● |  |  |  |
|  | 2 | 15 | 25 | ● | ● |  |  |  |  |
|  | 2 | 0 | 15 | ● | ● | ● |  |  |  |
|  | 2 | 0 | 16 | ● |  | ● |  |  | ● |
|  | 3 | 18 | 17 | ● | ● | ● | ● |  |  |
|  | 3 | 0 | 15 | ● | ● | ● | ● |  |  |
|  | 4 | 25 | 15 | ● | ● | ● |  |  |  |
|  | 4 | 20 | 23 | ● | ● | ● |  |  |  |
| **No** | 0 | 13 | 16 | ● |  |  |  |  |  |
|  | 0 | 6 | 0 | ● | ● |  | ● | ● |  |
|  | 1 | 20 | 0 | ● | ● |  |  | ● |  |
|  | 1 | 17 | 0 | ● | ● | ● | ● |  |  |
|  | 1 | 10 | 10 | ● | ● | ● |  |  |  |
|  | 2 | 20 | 20 | ● |  | ● | ● |  |  |
|  | 2 | 20 | 17 |  | ● |  |  |  |  |
|  | 2 | 13 | 0 | ● | ● |  | ● |  |  |
|  | 3 | 25 | 16 | ● | ● | ● | ● | ● |  |
|  | 4 | 31 | 25 | ● |  |  |  | ● |  |
|  | 4 | 18 | 16 |  | ● |  |  |  |  |
|  | 4 | 6 | 19 | ● |  | ● | ● |  |  |
