# Supplementary material for: C-Tb skin test to diagnose Mycobacterium tuberculosis infection in children and HIV-infected adults: A phase 3 trial
Source: PLoS One. 2018 Sep 24;13(9):e0204554. doi: 10.1371/journal.pone.0204554 (PMC6152999; doi:10.1371/journal.pone.0204554)
Supplement: S8 Table — Data are presented as n (%). Cut-points defining positive results were 5 mm (C-Tb) and 15 mm (TST). *Excluding 299 HIV-infected participants due to an uneven distribution in the various age groups. †Median (IQR) among responders with induration ≥1 mm. (DOCX) [file pone.0204554.s011.docx]

| **Age (years)** | **0-1** | **2-4** | **5-11** | **12-17** | **18-39** | **40-65** |
| --- | --- | --- | --- | --- | --- | --- |
| **N^*^** | 69 | 80 | 156 | 83 | 142 | 174 |
| **C-Tb** |  |  |  |  |  |  |
| **C-Tb ≥1 mm** | 16 (23.2) | 21 (26.6) | 63 (40.4) | 48 (57.8) | 86 (61.4) | 115 (66.9) |
| **C-Tb ≥5 mm** | 7 (10.1) | 14 (17.7) | 57 (36.5) | 42 (50.6) | 85 (60.7) | 113 (65.7) |
| **Not done** | 0 | 1 | 0 | 0 | 2 | 2 |
| **Median (mm)^†^** | 4  (2-15) | 18  (2-23) | 21  (12-32) | 22  (12-31) | 21  (16-26) | 21  (15-29) |
|  |  |  |  |  |  |  |
| **TST** |  |  |  |  |  |  |
| **TST ≥1 mm** | 26 (37.7) | 30 (37.5) | 67 (42.9) | 56 (67.5) | 115 (81.6) | 135 (78.5) |
| **TST ≥10 mm** | 14 (20.3) | 24 (30.0) | 61 (39.1) | 53 (63.9) | 108 (76.6) | 127 (73.8) |
| **TST ≥15 mm** | 5 (7.2) | 21 (26.3) | 48 (30.8) | 44 (53.0) | 88 (62.4) | 110 (64.0) |
| **Not done** | 0 | 0 | 0 | 0 | 1 | 2 |
| **Median (mm)^†^** | 10  (4-13) | 16  (10-20) | 20  (13-25) | 20  (15-25) | 18  (15-25) | 20  (16-28) |
|  |  |  |  |  |  |  |
| **QFT** |  |  |  |  |  |  |
| **QFT pos.** |  |  | 56 (36.1) | 41 (50.6) | 86 (64.7) | 105 (62.1) |
| **QFT ind.** |  |  | 23 (14.8) | 5 (6.2) | 9 (6.8) | 11 (6.5) |
| **Not done.** | 69 | 80 | 1 | 2 | 9 | 5 |
