# Supplementary material for: C-Tb skin test to diagnose Mycobacterium tuberculosis infection in children and HIV-infected adults: A phase 3 trial
Source: PLoS One. 2018 Sep 24;13(9):e0204554. doi: 10.1371/journal.pone.0204554 (PMC6152999; doi:10.1371/journal.pone.0204554)
Supplement: S9 Table — *McNemar’s test. Cut-point for TST was 15 mm. In an intention to diagnose principle, QFT indeterminate results were regarded as negative (arrows). †2 missing QFT. (DOCX) [file pone.0204554.s012.docx]

| **Control** | | **C-Tb** | |  |  | **Control** | | **C-Tb** | |  |
| --- | --- | --- | --- | --- | --- | --- | --- | --- | --- | --- |
|  |  | **Pos** | **Neg** | ∑ |  |  |  | **Pos** | **Neg** | ∑ |
| **TST** | **Pos** | 12 | 3 | 15 |  | **QFT** | **Pos** | 11 | 12 | 23 |
|  |  |  |  |  |  |  | **Ind** | 0 | 5↓ | 5 |
|  | **Neg** | 5 | 80 | 85 |  |  | **Neg** | 6 | 64 | 70 |
|  | ∑ | 17 | 83 | 100 |  |  | ∑ | 17 | 81 | 98^†^ |
|  | p^*^=0.7237;  κ=0.70 (0.51-0.90)  Concordance=92.0% | | | |  |  | p^*^=0.2386;  κ=0.44 (0.22-0.65) Concordance=81.6% | | | |

| **Control** | | **TST** | |  |
| --- | --- | --- | --- | --- |
|  |  | **Pos** | **Neg** | ∑ |
| **QFT** | **Pos** | 11 | 12 | 23 |
|  | **Ind** | 0 | 5↓ | 5 |
|  | **Neg** | 4 | 66 | 70 |
|  | ∑ | 15 | 83 | 98^†^ |
|  | p^*^=0.0801;  κ=0.48 (0.27-0.70)  Concordance=83.7% | | | |
