# Supplementary material for: C-Tb skin test to diagnose Mycobacterium tuberculosis infection in children and HIV-infected adults: A phase 3 trial
Source: PLoS One. 2018 Sep 24;13(9):e0204554. doi: 10.1371/journal.pone.0204554 (PMC6152999; doi:10.1371/journal.pone.0204554)
Supplement: S10 Table — *McNemar’s test. Cut-point for TST was 15 mm. †Including two with active TB. (DOCX) [file pone.0204554.s013.docx]

| **Asymptomatic**  **PTB contact** | | **C-Tb** | |  |  | **Symptomatic active + non-TB** | | **C-Tb** | |  |
| --- | --- | --- | --- | --- | --- | --- | --- | --- | --- | --- |
|  |  | **Pos** | **Neg** | ∑ |  |  |  | **Pos** | **Neg** | ∑ |
| **TST** | **Pos** | 23 | 5 | 28 |  | **TST** | **Pos** | 15^†^ | 11 | 26 |
|  | **Neg** | 5 | 54 | 59 |  |  | **Neg** | 6 | 116 | 122 |
|  | ∑ | 28 | 59 | 87 |  |  | ∑ | 21 | 127 | 148 |
|  | p^*^=0.7518;  κ=0.74 (0.58-0.89)  Concordance=88.5% | | |  |  |  | p^*^=0.3320;  κ=0.57 (0.39-0.75) Concordance=88.5% | | | |
